# The Flower Essential Oil of *Dalea mutisii* Kunth (Fabaceae) from Ecuador: Chemical, Enantioselective, and Olfactometric Analyses

**DOI:** 10.3390/plants9101403

**Published:** 2020-10-21

**Authors:** Gianluca Gilardoni, Mayra Montalván, Mariana Ortiz, Diego Vinueza, José Vinicio Montesinos

**Affiliations:** 1 Departamento de Química y Ciencias Exactas, Universidad Técnica Particular de Loja, Loja 1101608, Ecuador; msmontalvan@utpl.edu.ec (M.M.), 14mariana2008@hotmail.es (M.O.), jvmontesinos@utpl.edu.ec (J.V.M.); 2 Facultad de Ciencias, Escuela Superior Politécnica de Chimborazo, Lope de Armendariz, Riobamba 060106, Ecuador; drvinueza@espoch.edu.ec

**Keywords:** *Dalea mutisii*, essential oil, enantioselective analysis, GC–MS, GC–O, Ecuador

## Abstract

An essential oil was distilled with 0.25% yield from fresh flowers of *Dalea mutisii* Kunth, a native species mainly growing in the Andean region of Ecuador. A total of 50 compounds were identified, and most of them were quantified. The chemical composition was characterized by the prevalence of monoterpene hydrocarbons (>90%). Major components were α-pinene (42.9%), β-pinene (15.1%), β-phellandrene (12.6%), myrcene (6.7%), and (*Z*)-β-ocimene (5.4%). The essential oil was then submitted to enantioselective analysis, with a 2,3-diethyl-6-*tert*-butyldimethylsilyl-β-cyclodextrin-based capillary column. An enantiomeric excess was measured for (1*R*,5*R*)-(+)-α-pinene (91.6%), (1*R*,5*R*)-(+)-β-pinene (15.2%), (*R*)-(−)-α-phellandrene (4.8%), and (*R*)-(−)-β-phellandrene (88.8%), whereas (*R*)-(+)-limonene was enantiomerically pure. A gas chromatography–olfactometry (GC–O) analysis was additionally carried out on this pleasantly fragrant essential oil, following an aroma extract dilution analysis (AEDA) approach. Main odorants were α-pinene, β-pinene, α-phellandrene, and (*Z*)-β-ocimene, with dilution factors (FD) of 8, 4, 2, and 2, respectively.

## 1. Introduction

The study of natural products has been one of the leading ways of finding new molecules of biological interest since the beginning of 19th century. Currently, most European and many North American botanical species have been phytochemically investigated, resulting in a quite wide knowledge of their secondary metabolite composition and biological activity. As a consequence, despite these studies not yet being exhaustive, the discovery of new molecules within the European and North American flora is becoming more and more difficult. This is the reason why, during the next 30 years, the interest in looking for new metabolites shifted from temperate to tropical countries, where an impressive biodiversity, together with a more recent scientific history, makes phytochemical studies very important. Belonging to a group of 17 megadiverse countries [1], Ecuador is one of the most promising places in the world for carrying out these studies. For this reason, the authors have been investigating the endemic flora of Ecuador for many years, in order to contribute to the phytochemical knowledge of the Ecuadorian biodiversity [2].

Among natural products, a special interest exists in essential oils (EOs) [3,4,5,6,7,8,9,10,11], whose main property is their aroma. In fact, they are defined in the European Pharmacopoeia as “odorous products, usually of complex composition, obtained from a botanically defined plant raw material by steam distillation, dry distillation, or a suitable mechanical process without heating. Essential oils are usually separated from the aqueous phase by a physical process that does not significantly affect their composition” [12]. They are important commercial natural products, finding application as food aromas, industrial perfumes, and perfumery materials. Furthermore, EOs are often characterized by interesting biological activities. Nevertheless, even when their availability does not justify a commercial production, the EOs are mixtures of academic interest, representing the volatile fraction in the metabolic profile of a botanical species. The aim of the present study consisted of providing the chemical and sensory description of a new EO, with a view to possible applications thanks to its good yield and aromatic properties. 

*Dalea mutisii* Kunth is an Andean native shrub, belonging to the family Fabaceae, and it is widespread among Colombia, Ecuador, and Perú [13]. Nevertheless, Ecuador appears as the main area of diffusion, where the species has been described in the provinces of Azuay, Bolivar, Cañar, Carchi, Chimborazo, Cotopaxi, Imbabura, Loja, Pichincha, and Tungurahua [14]. This plant is also known with many botanical synonyms, such as *D. coerulea* (L. f.) Schinz and Tell., *D. astragalina* Kunth, *D. ayavacensis* Kunth, *D. caerulea* L. f., *D. cutervoana* Szyszylowicz, *D. longispicata* Ulbr., *Galega coerulea* L. f., *Parosela astragalina* (Kunth) Killip ex J.F. Macbr., *P. ayavacensis* (Kunth) J.F. Macbr., *P. coerulea* (L. f.) J.F. Macbr., *Tephrosia coerulea* (L. f.) Pers., and *D. caerullea* L. f. [13,14,15,16]. The shrubs grow at an altitude of 1000–4000 m above sea level [14].

This species is known with the popular name “*iso*” in Ecuador, where flowers are used to treat pneumonia [17]. In Colombia, they call it “*pispura*”, whose leaves and flowers are prepared as a decoction to ward off lice and fleas [16].

Many studies have been published on the phytochemistry of genus *Dalea*; however, most of them are related to non-volatile metabolites and their biological activities. On the other hand, only six articles described EOs distilled from this genus: *D. scoparia* A. Gray [18], *D. greggii* A. Gray [19], *D. lumholtzii* Robins. and Fern. [19], *D. carthagenensis* (Jacq.) J.F. Macbr. [20], *D. formosa* Torr. [21], *D. foliosa* (A. Gray) Barneby [22], and *D. strobilacea* Barneby [23], with the latter being the corresponding species. With regard to *D. mutisii*, only two studies were found on its nonvolatile metabolites, describing prenylated flavonoids as the main compounds [24,25]. However, to the best of the authors’ knowledge, no chemical information has been published about its volatile fraction. Hence, in this report, we describe for the first time the chemical and the enantiomeric composition of an EO distilled from the flowers of *D. mutisii*. Since this EO was characterized by an intense and pleasant woody–resinous fragrance, the study was complemented with an olfactometric evaluation of its aromatic profile, carried out by gas chromatography–olfactometry (GC–O).

## 2. Results

### 2.1. Chemical Analysis and Physical Properties

The distillation of the fresh flowers of *D. mutisii* afforded an EO, with a mean distillation yield of 0.25% (*w/w*). The mean relative density of the EO was 0.891 g/cm^3^, whereas the mean refraction index was 1.4773. In the chemical analysis, 50 compounds were identified and 35 were quantified, corresponding to 98.3% of the whole sample mass. The EO mainly constituted monoterpene hydrocarbons, α-pinene (42.9%), β-pinene (15.1%), β-phellandrene (12.6%), myrcene (6.7%), and (Z)-β-ocimene (5.4%). The monoterpene fraction contributed to 91.8% of the whole EO. Only traces of one oxygenated monoterpene (cryptone) were detected. On the other hand, many sesquiterpenes and sesquiterpenoids were identified, being 4.8% and 1.6% of the respective sesquiterpene hydrocarbon and oxygenated fractions. Small amounts of phenylpropanoids and aliphatic esters were also observed, with (*E*)-*iso*-amyl cinnamate as the only quantifiable one (0.1%). The complete chemical analysis is shown in Table 1.

### 2.2. Enantioselective Analysis

**Figure 1:**
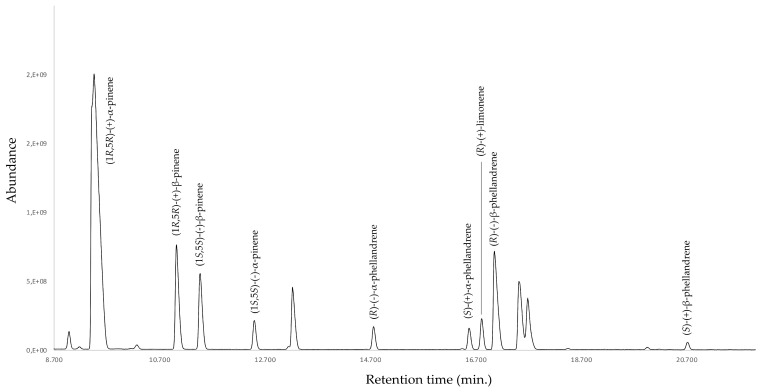
Enantioselective chromatogram of *D. mutisii* EO with 2,3-diethyl-6-*tert*-butyldimethylsilyl-β-cyclodextrin column.

The enantioselective analysis [28,29] was performed with a capillary column, using 2,3-diethyl-6-*tert*-butyldimethylsilyl-β-cyclodextrin as a chiral selector. A total of four monoterpene enantiomeric pairs were identified, calculating the respective enantiomeric distribution and enantiomeric excess (ee). Only (*R*)-(+)-limonene was identified as an enantiomerically pure component. The complete enantioselective analysis is presented in Table 2 and Figure 1, where the baseline separation of all the identified enantiomers can be observed.

### 2.3. Olfactometric Analysis

**Figure 2:**
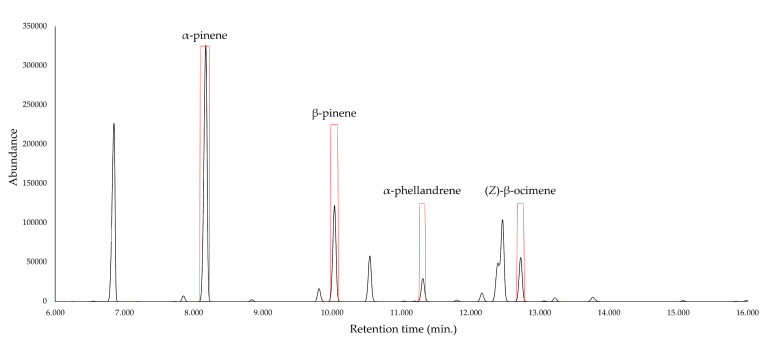
AEDA aromagram of *D. mutisii* EO with DB-5ms column.

The olfactometric analysis was carried out through gas chromatography–olfactometry (GC–O) [30], following an aroma extract dilution analysis (AEDA) protocol [31,32,33]. Four olfactory relevant compounds were detected, with α-pinene being the principal sensory component of the EO, with a dilution factor (FD) of 8. The results of the GC–O analysis are represented in Table 3 and Figure 2.

## 3. Discussion

The EOs obtained from plants of genus *Dalea*, whose chemical composition is described in the literature, are all characterized by a monoterpene composition, where two/three compounds represented the major constituents. The volatile fraction distilled from *D. mutisii* presented the same feature, with α-pinene, β-pinene, and β-phellandrene representing more than 70% of the total composition. The most similar EO was that described for *D. strobilacea* [23], where α-pinene and β-phellandrene were major components, and β-pinene was the most abundant. However, the relative abundances of α-pinene and β-phellandrene were inverted, being 42.9% and 12.6%, respectively, in *D. mutisii* and 17.7% and 43.5% in *D. strobilacea*. Furthermore, in the latter, myrcene slightly exceeded β-pinene. In *D. carthagenensis* EO, the main components were β-pinene (14–16%) and (*E*)-β-ocimene (12–36%) [20]; in *D. scoparia* (*Psorothamnus scoparius*), they were *p*-cymene (14.0%) and γ-terpinene (22.3%); in *D. formosa*, major constituents were α-pinene (31.7%) and camphene (8.4%). Despite the differences, all these EOs were characterized by monoterpene hydrocarbons as the main components. A quite different composition was described for the volatile fraction of *D. foliolosa*, whose EO presented oxygenated monoterpenoids as the major components. In this case, linalool (10–17%) and cryptone (22–30%) were the main constituents, together with a very relevant sesquiterpene fraction accounting for 10–23% of the whole amount. In this respect, caryophyllene oxide (5–15%) and α-cadinol (1–6%) were the most abundant sesquiterpenoids [22].

With regard to the sensory properties of *D. mutisii* EO, a strong and pleasant woody–resinous odor motivated us to study the aromatic profile using gas chromatography–olfactometry. An AEDA evaluation of the mixture was performed, which afforded four components as mainly responsible for the olfactory properties. According to this analysis, α-pinene, β-pinene, α-phellandrene, and (*Z*)-β-ocimene were the aroma-determining components, with a dilution factor (FD) of 8, 3, 2, and 2, respectively. In order to obtain a more comprehensive information on the chemical composition in general and the aromatic profile in particular, the study was complemented with the enantioselective analysis of the monoterpene fraction. In fact, it is well known that the enantiomeric composition of a chiral mixture influences its biological properties, including sensory perception [34]. In *D. mutisii* EO, α-pinene, β-pinene, α-phellandrene, and β-phellandrene were present as enantiomeric mixtures, whilst (*R*)-(+)-limonene was enantiomerically pure. The enantiomeric excess of β-pinene and α-phellandrene was close to the racemic mixture, whilst, for (1*R*,5*R*)-(+)-α-pinene and (*R*)-(−)-β-phellandrene, it was 91.6% and 88.8%, respectively. All these results were consistent with the perceived aroma of the whole EO.

Despite the different focus of the present study, some consideration could be afforded to the chemical composition and the biological properties of this EO. In order to formulate some consistent hypotheses, we should compare our volatile fraction with that from *D. strobilacea* [23], whose chemical composition is the most similar among the EOs described in literature for this genus. *D. strobilacea* is an aromatic plant, growing wild in the highlands from Peru to Chile. It is used in traditional medicine as “*hierba de chil*”, in the form of a decoction, for treating gastrointestinal disorders. No application has been described that correlates the traditional use of this plant with that of *D. mutisii*; however, the EO from *D. strobilacea* was submitted to some interesting antibacterial essays. In particular, for its minimum inhibitory concentration (MIC), it resulted very active against *Enterococcus faecalis* (MIC = 7.81 μg/mL vs. >125 μg/mL for vancomycin) and not very active against *Klebsiella pneumoniae* (MIC = 59.5 μg/mL vs. 15.4 μg/mL for vancomycin) [23]. These results are consistent with the traditional use of *D. strobilacea* but do not support the main traditional use of *D. mutisii* against pneumonia. Four hypotheses can be formulated in this case: (1) the EOs are similar in composition but not identical, which may explain the difference; (2) the enantiomeric composition of the active components could be different; (3) the anti-pneumonia activity of *D. mutisii* could be due to its nonvolatile fraction; (4) the traditional use of *D. mutisii* to treat pneumonia could be scientifically unsupported. Nevertheless, all these considerations are inconclusive in the face of a lack of direct bioactivity-based evidence, merely constituting some working hypotheses.

## 4. Materials and Methods

### 4.1. Plant Material

The flowers of *D. mutisii* were collected in 2018 at Riobamba (2879 m above sea level) and La Asunción (3083 m above sea level), corresponding to coordinates of 1°39′29″ south (S), 78°40′35″ west (W) and 1°38’10.743” S, 78°44’15.352” W, respectively. One sample (about 350 g) was provided from Riobamba and two samples (about 435 g each) from La Asunción. The plant material was collected by some of the authors under permission N° 001-IC-FLO-DBAP-VS-DRLZCH-MA, granted by the Ministry of Environment of Ecuador (MAE), and distilled immediately after collection. The species was identified by botanist Jorge Caranqui of the Escuela Superior Politécnica de Chimborazo (ESPOCH), whereas a voucher specimen was deposited at the herbarium of the same university with code ESPOCH-1047.

### 4.2. Distillation of the EO and Sample Preparation

A total of three samples of pure EO were obtained by preparative steam distillation of each fresh sample of plant material. Each distillation was carried out for 3 h, inside a stainless-steel Clevenger-type apparatus. After recovery of the organic layer, which spontaneously separated from water, the EO was dried over anhydrous sodium sulfate and immediately stored in amber vials at −15 °C. For all GC injections, about 10 mg of EO were exactly weighted and diluted with 1 mL of cyclohexane, containing *n*-nonane as internal standard (0.7 mg/mL).

### 4.3. Qualitative Chemical Analysis

The qualitative analysis was run with a gas chromatography–mass spectrometry (GC–MS) system, consisting of an Agilent Technologies gas chromatograph 6890N, coupled with a quadrupole Mass Spectrometry Detector (MSD) 5973 (Santa Clara, CA, USA). The MSD was operated in SCAN mode and electronic ionization (70 eV), set at a mass range detection of 35–350 *m/z*. The MS transfer line temperature was set at 280 °C, while the ion source temperature was set at 200 °C. The gas chromatograph was equipped with a nonpolar stationary phase capillary column DB-5ms (5% phenyl-methylpolysiloxane, 30 m length, 0.25 mm internal diameter, and 0.25 μm film thickness; J & W Scientific, Folsom, CA, USA). The GC–MS analyses were performed as follows: the carrier gas was helium, set at a constant flow rate of 1 mL/min. The injection volume was 1 μL, with the injector operated in split mode (split ratio of 40:1) at the temperature of 250 °C. The elution was conducted from 50 °C (1 min) to 250 °C (10 min) at a gradient rate of 3 °C/min.

The EO components were identified by comparing both their linear retention indices (LRIs), calculated according to van den Dool and Kratz [26], and their mass spectra to those reported in literature (see Table 1). The linear retention indices were calculated using the homologous series of linear alkanes from *n*-nonane to *n*-pentacosane (C_9_ purity 99% from BDH, Dubai, UAE and C_10_-C_25_ purity 99% from Sigma-Aldrich, St. Louis, MO, USA). The identification of major components (>5%) was confirmed by injection of pure reference standard samples. All solvents and standards used in this study (analytical grade, purity > 99%) were purchased from Sigma-Aldrich.

### 4.4. Quantitative Chemical Analysis

The quantitative analysis was carried out with the same GC system as the qualitative one, coupled with a flame ionization detector (FID), and equipped with an Agilent Technologies 7683 series autoinjector (Little Falls, DE, USA).

The instrumental conditions were the same as the qualitative analyses but with a different thermal program: 50 °C for 1 min, a first thermal gradient to 180 °C at a rate of 3 °C/min, and then a second gradient to 250 °C at a rate of 15 °C/min. At the end, the oven temperature was kept at 250 °C for 15 min. The FID was alimented as follows: hydrogen flow 30 mL/min, air flow 300 mL/min. The temperature of the detector was set at 250 °C. The quantitative composition was obtained by using relative response factors (RRFs), calculated on the basis of the combustion enthalpy [35,36]. The RRFs were directly applied to the internal standard peak, which served for both normalization and quantification. The original method was modified, since *n*-nonane instead of methyl octanoate was used as internal standard. In order to carry out the quantitative analysis, the preparation described in Section 4.2 was applied twice to each pure EO, affording a total of six analytical samples. The quantitative results (Table 1) were obtained as mean values and standard deviations.

### 4.5. Enantioselective GC Analysis

The enantioselective analysis was carried out using the same GC–MS system described for the qualitative one, configured with a 2,3-diethyl-6-*tert*-butyldimethylsilyl-β-cyclodextrin enantioselective column (25 m × 0.25 mm × film thickness 0.25 µm from Mega, Legnano, Italy).

The following thermal program was used: 50 °C, held for 5 min, rising to 220 °C at a rate of 2 °C/min, and kept at this temperature for 5 min. The elution order was established according to literature, where enantiomerically pure standards were injected in the same column and conditions [3,10].

### 4.6. GC–O Analysis

The GC–O analyses were carried out with the same GC–FID system described for the quantitative ones, coupled to a sniffing port device model ODP 3, from Gerstel GmbH & Co.KG., Mülheim an der Ruhr, Germany. The GC–O system was configured with a 50% split ratio between sniffing port and detector. The olfactometric evaluations were performed by a panel of four trained people, presenting no anosmia for common monoterpenes and following an AEDA approach [31,32,33]. The samples were prepared as solutions of the EO in cyclohexane, at the concentration of 200 μL/mL, corresponding to a dilution factor (FD) of 1. The injection volume was 1 μL. The AEDA method was applied by acting on the split ratio, according to the following sequence: splitless, 1:1, 2:1, 3:1, 4:1, 5:1, and 6:1. After a preliminary qualitative screening at the highest concentration, the sniffing procedure of each panelist was carried out until 16 min. The acceptance criteria for the detected odors and FD values was that each perception had to be confirmed by at least three panelists in at least two following dilutions or, alternatively, once by all the panelists at the same dilution. During the analysis, the panelists were asked to give a descriptor for each perceived odor, utilizing the adjectives commonly used for terpenes and terpenoids. 

## 5. Conclusions

The flowers of *D. mutisii* Kunth produce an EO, characterized by a pleasant woody–resinous odor and a quite good yield of 0.25% (*w/w*). The chemical composition is dominated by monoterpenes, which contribute to more than 90% by weight. The main chiral components of the EO are present as enantiomeric pairs, except for the case of the enantiomerically pure (*R*)-(+)-limonene. With regard to the olfactory profile, four monoterpene hydrocarbons appear to be determinant. In fact, α-pinene, β-pinene, α-phellandrene, and (*Z*)-β-ocimene are the main odorous constituents, in order of decreasing importance. The descriptors assigned to these compounds during the GC–O analysis are consistent with the perceived fragrance of the whole EO.

## Figures and Tables

**Table 1:** Chemical analysis of *Dalea mutisii* essential oil (EO) using a DB-5ms column.

No.	RT ^1^ (min)	LRI ^2^		Components	Quantitative Analysis	
		Calculated ^3^	Reference ^4^		% ^5^	Σ ^6^
1	7.73	921	926	tricyclene	Trace	-
2	7.85	924	930	α-thujene	0.9	0.14
3	8.18	932	939	α-pinene	42.9	6.07
4	8.84	947	954	canfene	0.3	0.04
5	9.81	970	975	sabinene	1.8	0.29
6	10.04	976	979	β-pinene	15.1	0.19
7	10.55	988	990	myrcene	6.7	1.18
8	11.2	1003	1004	*p*-mentha-1(7),8-diene	Trace	-
9	11.31	1005	1002	α-phellandrene	1.5	1.28
10	11.81	1015	1024	*p*-cymene	0.1	0.08
11	12.17	1023	1026	*o*-cymene	0.9	0.83
12	12.4	1028	1029	limonene	2.3	1.46
13	12.46	1029	1029	β-phellandrene	12.6	3.42
14	12.73	1035	1037	(*Z*)-β-ocimene	5.4	1.93
15	13.22	1045	1050	(*E*)-β-ocimene	0.5	0.26
16	13.77	1056	1059	ϒ-terpinene	0.7	0.13
17	15.07	1083	1088	*p*-mentha-2,4(8)-diene	0.1	0.10
18	15.83	1099	1100	*iso*-pentyl-2-menthyl butanoate	Trace	-
19	15.98	1102	1104	2-methyl butyl-*iso*-valerate	Trace	-
20	16.06	1104	1104	2-(*E*)-hexenyl propanoate	Trace	-
21	19.97	1185	1185	cryptone	Trace	-
22	27.32	1345	1351	α-cubebene	0.1	0.04
23	28.56	1373	1376	α-copaene	0.2	0.10
24	28.91	1381	1378	(*E*)-methyl cinnamate	Trace	-
25	29.1	1385	1388	β-cubebene	0.1	0.02
26	29.89	1404	1409	α-gurjunene	Trace	-
27	30.09	1408	1407	longifolene	Trace	-
28	30.42	1416	1408	(*Z*)-β-caryophyillene	0.4	0.29
29	31.03	1431	1434	α-*trans*-bergamotene	Trace	-
30	31.19	1435	1432	β-copaene	0.1	0.07
31	31.91	1452	1454	α-humulene	0.1	0.05
32	32.09	1456	1460	*allo*-aromandendrene	Trace	-
33	32.73	1472	1466	*cis*-muurola-4(14),5-diene	0.1	0.11
34	32.97	1478	1479	ϒ-muurolene	Trace	-
35	33.45	1489	1493	α-zingiberene	1.6	0.84
36	33.59	1493	1493	*trans*-muurola-4(14),5-diene	0.1	0.07
37	33.71	1495	1500	α-muurolene	1,0	0.46
38	34.00	1503	1505	(*E,E*)-α-farnesene	0.4	0.30
39	34.27	1509	1513	ϒ-cadinene	Trace	-
40	34.39	1512	1512	δ-amorphene	0.1	0.06
41	34.49	1515	1523	δ-cadinene	0.1	0.07
42	34.61	1518	1522	*trans*-calamenene	0.3	0.46
43	35.05	1529	1534	*trans*-cadina-1,4-diene	0.1	0.05
44	36.98	1578	1575	zierone	0.2	0.14
45	37.85	1600	1600	guaiol	1.1	0.38
46	38.11	1607	1607	dodecyl acetate	Trace	-
47	38.74	1624	1619	1,10-di-*epi*-cubenol	Trace	-
48	39.29	1639	1640	*epi*-α-cadinol	0.2	0.18
49	39.77	1652	1658	valerianol	0.1	0.15
50	43.06	1744	1741	(*E*)-*iso*-amyl cinnamate	0.1	0.09
				*Monoterpene hydrocarbons*	*91.8*	
				*Oxygenated monoterpenes*	*Trace*	
				*Sesquiterpene hydrocarbons*	*4.8*	
				*Oxygenated sesquiterpenes*	*1.6*	
				*Other compounds*	*0.1*	
				*Total*	*98.3*	

^1^ Retention time; ^2^ linear retention index; ^3^ according to van den Dool and Kratz [26]; ^4^ according to [27]; ^5^ % by weight referred to whole sample; ^6^ standard deviation; trace < 0.1%.

**Table 2:** Enantioselective analysis of some chiral constituents of *D. mutisii* EO with 2,3-diethyl-6-*tert*-butyldimethylsilyl-β-cyclodextrin column.

RT ^1^ (min)	LRI ^2^	Enantiomers	Enantiomeric Distribution (%)	*ee*^3^ (%)
9.46	932	(1*R*,5*R*)-(+)-α-pinene	95.8	91.6
12.50	987	(1*S*,5*S*)-(−)-α-pinene	4.2	
11.03	960	(1*R*,5*R*)-(+)-β-pinene	57.6	15.2
11.47	968	(1*S*,5*S*)-(−)-β-pinene	42.4	
14.76	1026	(*R*)-(−)-α-phellandrene	52.4	4.8
16.58	1057	(*S*)-(+)-α-phellandrene	47.6	
16.81	1061	(*R*)-(+)-limonene	100.0	100.0
17.06	1065	(*R*)-(−)-β-phellandrene	94.4	88.8
20.72	1127	(*S*)-(+)-β-phellandrene	5.6	

^1^ Retention time; ^2^ linear retention index; ^3^ enantiomeric excess.

**Table 3:** Olfactometric analysis and sensory descriptors of *D. mutisii* EO with DB-5ms column.

LRI ^1^	AEDA ^2^ (FD) ^3^	Components	Descriptor
932	8	α-pinene	Woody
976	4	β-pinene	Woody
1005	2	α-phellandrene	Herbaceous
1035	2	(*Z*)-β-ocimene	Sweet

^1^ Linear retention index; ^2^ aroma extract dilution analysis; ^3^ dilution factor.
